# ﻿*Viciamingyueshanensis* (Fabeae, Papilionoideae, Fabaceae), a new species from western Jiangxi, China

**DOI:** 10.3897/phytokeys.187.71960

**Published:** 2021-12-13

**Authors:** Zhi-Yong Xiao, Xiao-Chun Li, Ying Luo, Chuan-Sheng Zeng, Bang-Gui Qiu, Fu-Liang Cao

**Affiliations:** 1 Yichun Academy of Sciences, Yichun, Jiangxi 336000, China; 2 Central South University of Forestry and Technology, Changsha, Hunan 410004, China

**Keywords:** Flora of China, Leguminosae, new taxon, taxonomy, *Vicia*

## Abstract

*Viciamingyueshanensis*, a new species from the Mingyue Mountain Region of western Jiangxi, China, is described and illustrated. It is a perennial climbing liana that always links to riparian woods. A morphological comparison indicated that the new species is closely similar to *Viciataipaica* K. T. Fu and *Viciadichroantha* Diels; however, it differs from the other two species by several salient characters, such as plant indumentum, stipule shape, corolla colour, bractlet shape and calyx shape. Photographs, a preliminary conservation assessment, table of morphological characters and distribution map comparing this new species to two morphologically-similar species are also provided.

## ﻿Introduction

The genus *Vicia* Linn. (Fabeae, Papilionoideae, Fabaceae) comprises about 180–200 annual or perennial herbaceous species, which are mainly distributed throughout the temperate regions of Europe, Asia, Africa, North, and South America (Kupicha 1976; Gunn 1979; Hanelt and Mettin 1989). This genus is widely distributed throughout China. Till now, 40 species of *Vicia* have been reported in China (Xia 1996; Bao and Turland 2010). Over many years, due to its high biological yield and high content of the crude protein within a short growth period, *Vicia* is considered with potential value as forage and it is extensively planted globally (Maršalkienė 2016).

During field surveys carried out in May 2019, a population of a perennial *Vicia* species was discovered in the Mingyue Mountain Region (Jiangxi Province, China). Detailed comparisons showed that the specimens and living plant materials were different from the type of specimens and protologues of some related known *Vicia* species. Moreover, the shapes of its leaf and rhizomes were most similar to those of *Viciataipaica* K. T. Fu and *Viciadichroantha* Diels. The three species are perennial herbs with branched stems that climb by means of tendrils on the ends of their paripinnate leaves. However, the new species can easily be distinguished from the latter two by several morphological characters (Table 1).

On the basis of careful investigations of herbarium specimens and living material and after the observation and cultivation in two years, the new species *Viciamingyueshanensis* is described in this paper. The genus Vicia is divided into two large subgenera, subgen. Cracca and subgen. Vicia. Due to the perennial herbaceous and climbing habit of the new species, as well as the presence of tendrils, it belongs to subgen. Cracca.

## ﻿Materials and methods

This study was mainly based on field surveys, the detailed examinations of herbarium specimens and literature. Herbarium specimens were examined in **PE**, **KUN** and **JJF** and from online specimen images from the International Plant Name Index (IPNI, https://www.ipni.org), Jiangxi Virtual Herbarium (JVH, http://site.nsii.org.cn/api/site.ashx?id=JXVH&a=app&app=VHForeword) and the Chinese Virtual Herbarium (CVH, https://www.cvh.ac.cn/index.php), National Specimen Information Infrastructure (NSII, http://www.nsii.org.cn/2017/home-en.php) and NYBG Steere Herbarium (http://sweetgum.nybg.org/science/vh/). Specimens collected from the field were deposited at the CSFI and NF. Detailed observations and measurements of the collected individuals were undertaken and micromorphological features were analysed using a Leica MZ16 stereomicroscope.

## ﻿Taxonomic treatment

### ﻿ Vicia mingyueshanensis

Taxon classificationPlantaeFabalesFabaceae

Z.Y.Xiao & X.C.Li sp. nov.

06C674F6-4328-5ADD-9BCD-F02DE8C52461

urn:lsid:ipni.org:names:77234378-1

**Figure 1.**
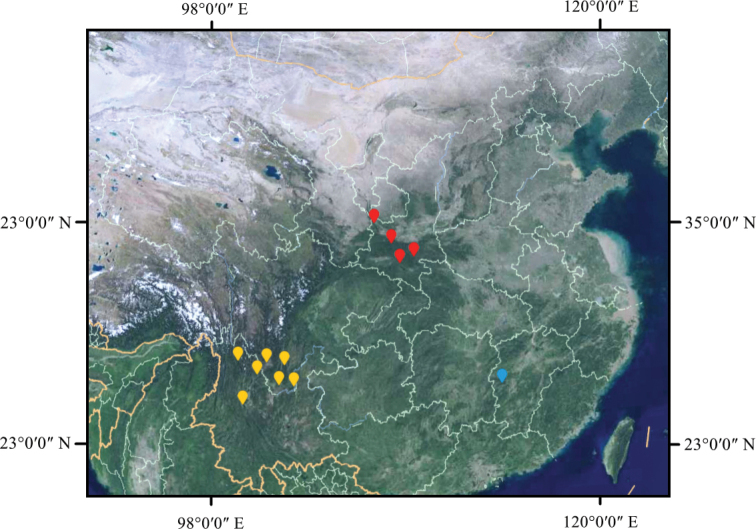
Distribution map of *Viciamingyueshanensis* (blue dots) and its closest similar species Viciataipaica (red dots) and Viciadichroantha (yellow dots).

**Figure 2.**
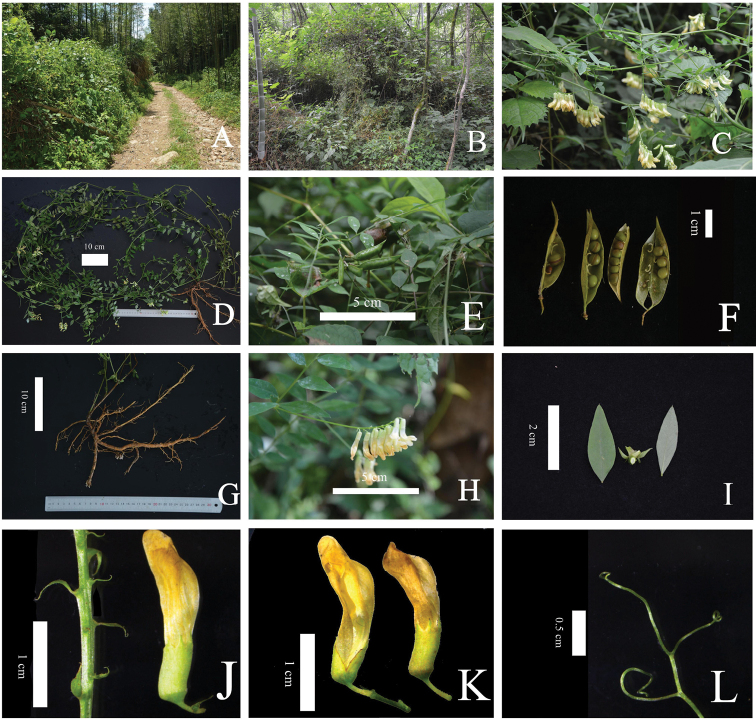
*Viciamingyueshanensis***A, B** habitat **C** habit **D** the whole plant **E** pods **F** pods and seeds **G** root **H** inflorescences **I** leaflets and stipules **J** bractlet **K** calyx **L** tendrils.

Figures 1 , 2 , Table 1

#### Type.

China. Jiangxi Province, Yichun County, Hongjiang Township, Dongnan Village, under bamboo forests, beside the river ditch, 328 m elevation, 8 May 2019, *Z.Y. Xiao & X.C.Li*, CSFI076074 (holotype: CSFI; isotypes: NF).

#### Diagnosis.

Sepal lobes and bractlets, completely glabrous. Most similar to *Viciataipaica*, but differs from it by its hastate or lanceolate stipules and subulate bractlets (stipules semi-ovate or lanceolate and bractlets absent in *Viciataipaica*). Similar to *Viciadichroantha* as well, but differs from it by the light yellow or dull orange colour of the corolla and subulate bractlet (yellow, dark yellow or dull orange corolla, marked purple at apex of standard and braclets, absent in *Viciadichroantha*). The new species is restricted to western Jiangxi Province (Figs 1, 2, Table 1).

#### Description.

Perennial herb, strongly climbing, 0.5–1.8 m tall, glabrous throughout. Root robust, woody, branched, well-developed in depth. Stems flexuous, subquadrangular, striate, branched. Leaves paripinnate, 8–15 cm (excluding the tendril), with 4–6 pairs alternate leaflets, provided with a terminal twining tendril, 2–3 branched; leaflets elliptic to ovate-oblong, margin entire, not toothed, papery, 2.3–3.8 cm long, 0.7–1.5 cm wide, broadly cuneate or suborbicular at the base, mucronulate at the apex, subsessile or shortly petiolulate (to ca. 1 mm long), lateral veins 7–12 paired. Stipules opposite, unequal, margin entire, hastate or lanceolate, 0.4–0.7 × 0.2–0.3 cm. Racemes 10–20 flowered, shorter or nearly as long as the subtending leaves, with peduncle up to 4–8 cm long. Flowers slightly pendent, 1.6–2.0 cm long, bractlet, subulate, 0.2–0.3 × 0.1 cm. Calyx membranaceous, obliquely campanulate, 0.4–0.5 cm long, tubular, gibbous at the base, zygomorphic, with 5 lateral teeth acute, some calyces are cleft. Corolla light yellow or dull orange, standard with 1.3–1.4 × 0.4–0.5 cm, subequalling to wings and keels, apex retuse. Staminal tube 1.2–1.4 cm long, vexillary staminal filament free, anther greenish-yellow. Ovary 0.5–0.6 cm long, with 4–6 ovules. Style geniculate at the base, cylindrical, 0.3 cm long, evenly hairy under the stigma. Pod stipitate, falcate, often apiculate, smooth, 3.0–3.5 × 0.3 cm. Seeds 4–6, oblate-spheroid, brown-green, 0.3–0.4 × 0.3 cm, hilum circumlinear, up to the middle of the circumference long.

#### Phenology.

Flowering time from May to early June; fruiting in July and defoliation from late July to early August.

**Table 1.** Detailed comparison of *Viciamingyueshanensis* and its two morphologically-similar species.

Characters	*Viciamingyueshanensis*	*Viciataipaica*	*Viciadichroantha*
Plant indumentum	totally glabrous	totally glabrous	densely hairy
Stem height (m) and appearance	0.5–1.8, relatively thin decumbent	0.6–1.0, relatively thick decumbent	0.6–2.0, relatively thin erect
Leaf length (cm), tendril excluded	8–15	8–12	7–16
Leaflet pairs per leaf	4–6	3–5	4–6
Leaflet shape	elliptic to ovate-oblong	elliptic to ovate-oblong	linear to linear-lanceolate
Leaflet size (cm)	2.3–3.8 × 0.7–1.5	1.3–5.0 × 0.6–1.5	2.5–5.0 × 0.6–0.9
Stipule shape and size (cm)	hastate or lanceolate, opposite, unequal, margins entire, 0.4–0.7 × 0.2–0.3	semi-ovate or lanceolate, margin entire, 0.5–0.9 long	fan-shaped or lanceolate, margin 2–3 toothed
Raceme (number of flowers)	10–20	5–15	20–25
Corolla colour	light yellow or dull orange	yellow or brown-yellow	yellow, dark yellow or dull orange, marked purple at the apex of standard
Bractlet shape	subulate	absent	absent
Calyx shape	5 lateral teeth acute, some calyces are cleft	shortly and unequally toothed	5 lateral teeth acute, hairy
Seed colour and size (cm)	brown-green	oblong	oblate-spheroid
	0.3–0.4 × 0.3	0.3–0.4 × 0.2	0.3–0.4 × 0.4
Seed numbers	4–6	2–5	2–4

#### Etymology.

The species epithet is derived from the name of the mountain range (Mingyueshan) where the species had been discovered.

#### Vernacular name.

The Chinese name ‘明月山野豌豆’ (Ming Yue Shan Ye Wan Dou)

#### Distribution and habitat.

*Viciamingyueshanensis* is only known in western Jiangxi Province, Yichun County, Hongjiang Town, Dongnan Village, Mingyue Mountain Region, located in an open area of *Phyllostachysedulis* J. Houzeau forests with *Castanopsistibetana* Hance and *Lithocarpuslitseifolius* (Hance) Chun as associated tree species. The observed population is very small, with fewer than 200 plants growing along roadsides and ditches, accompanied by *Oreocnidefrutescens* (Thunb.) Miq. and *Rubustephrodes* Hance. Elevation is 300–650 m above sea level.

#### Preliminary conservation assessment.

*Viciamingyueshanensis* is currently only known from a small population in a habitat that is subject to logging and disturbance, thus, it is very rare and distributed in a few patches. On the basis of our field observations, this species is represented by no more than 200 large and mature individuals, along a road where bamboo was being cut. Due to its rarity and a low number of individuals, *Viciamingyueshanensis* is considered to be **Critically Endangered** (**CR, B1**), according to the IUCN (2019).

## Supplementary Material

XML Treatment for Vicia mingyueshanensis
